# Do Statins Have a Positive Impact on Patients with Coronary Microvascular Dysfunction on Long-Term Clinical Outcome? A Large Retrospective Cohort Study

**DOI:** 10.1155/2019/4069097

**Published:** 2019-03-17

**Authors:** Wen-hao Luo, Yang Guo, Jie-wu Huang, Pei-dong Zhang

**Affiliations:** ^1^The Second School of Clinical Medicine, Southern Medical University, Guangzhou, China; ^2^Department of General Surgery, Peking Union Medical College Hospital, Chinese Academy of Medical Sciences and Peking Union Medical College, Beijing 100730, China; ^3^Department of Neurology, Zhujiang Hospital, Southern Medical University, Guangzhou, China; ^4^Department of Cardiology, Zhujiang Hospital, Southern Medical University, Guangzhou, China

## Abstract

**Objectives:**

To investigate the influence of statins on major adverse cardiovascular events (MACE) in patients with coronary microvascular dysfunction (CMVD).

**Participants:**

23,494 patients who received coronary angiography (CAG) were included. Thrombolysis in Myocardial Infarction, Myocardial Perfusion Grading (TMPG), a useful angiographic method, was used to evaluate CMVD.

**Results:**

Using multivariate analysis, NYHA III/IV (HR, 1.44; 95% CI, 1.03-2.01; P=0.031), PCI history (HR, 3.69; 95% CI, 2.57-5.31; P<0.001), TG (HR, 1.15; 95% CI, 1.06-1.26; P=0.001), creatinine (HR, 1.00; 95% CI, 1.00-1.01; P<0.001), cTnT (HR, 0.98; 95% CI, 0.96-0.99; P<0.001), heart rate (HR, 0.98; 95% CI, 0.97-0.99; P=0.001), *β*-blocker (HR, 0.68; 95% CI, 0.51-0.91; P=0.008), aspirin (HR, 0.38; 95% CI, 0.24-0.61; P<0.001), and statins (HR, 0.33; 95% CI, 0.19-0.60; P<0.001) significantly correlated with reduced MACE in CMVD patients. In subgroups analysis, statins decreased MACE overall (HR, 0.33; 95% CI, 0.19-0.59; P<0.001) and in CMVD patients with smoking history (HR, 0.64; 95% CI, 0.43-0.93; P=0.014), diabetes (HR,0.27; 95% CI,0.12-0.61; P=0.002), hypertension (HR, 0.10; 95% CI, 0.03-0.36; P=0.001), and hypertension and diabetes (HR, 0.09; 95% CI, 0.014-0.53; P=0.008).

**Conclusion:**

Statins could reduce MACE in patients with CMVD.

## 1. Introduction

Approximately 40% of patients who underwent coronary angiography for chest pain had nearly normal coronary angiography results but were considered to have coronary microvascular dysfunction (CMVD) [1–4]. Coronary microvasculature refers to the microcirculatory system in heart, consisting of arterioles, capillaries, and venules, which can regulate blood flow resistance and maintain function of myocardial cells and play significant role in manage coronary perfusion. Increasing evidence has shown that structural or functional coronary microvascular abnormality may lead to myocardial ischemia and cause CMVD and major adverse cardiovascular events (MACE). There are numerous methods to diagnose CMVD, such as thrombolysis in myocardial infarction (TIMI) frame count (TFC), TIMI myocardial blush grading (TMBG), and myocardial blush grade (MBG), which indirectly evaluate microvascular function through assessment of coronary circulation resistance or myocardial perfusion. Recently, new methods have been developed to assess CMVD, such as index of microvascular resistance (IMR). However, the relationship between IMR measurements and cardiovascular events remains unclear, and large sample and multicenter followup studies were needed to determine the feasibility of IMR. Among methods to assess postreperfusion CMVD, angiography provides low-cost, rapid, real-time evaluations. Extended contrast washout from infarcted myocardium represents a typical angiographic marker of microvascular impairment, which led to establishment of Thrombolysis in Myocardial Infarction, Myocardial Perfusion Grading (TMPG) [5], a widely-used angiographic assessment for microvascular perfusion with good accuracy for MACE diagnosis in clinical trials [6, 7].

Several cofactors including age, hypertension, smoking history, diabetes, and increased C-reactive protein levels are associated with poor coronary blood flow [8, 9]. Although several methods have been used to reduce disease and subsequent death in patients with CMVD [10], identification of predictors of hazard rate could help guide clinical treatment course.

2013 ESC guidelines on management of stable coronary artery disease recommend *β*-blockers as a first-line treatment for CMVD, and calcium antagonists are recommended if *β*-blockers do not result in sufficient symptomatic benefit or are not tolerated (Class I Level B). ESC guidelines also recommended that all patients with CMVD should receive secondary preventive medications such as statins [11]. However, evidence for the relationship between CMVD and MACE was unclear. Furthermore, it is also unclear whether statin treatment plays a significant role in decreasing MACE in patients with CMVD. This study aimed to assess the impact of statin treatment in CMVD patients.

## 2. Materials and Methods

This study consisted of patients in the electronic clinical research database at ZhuJiang hospital, a comprehensive teaching and educational hospital specializing in integrating clinic, education, and research in the GuangZhou of China. In this study, we performed a retrospective population-based cohort analysis of patients who underwent CAG or PCI between January 2007 and March 2018. The primary goal of the program was to evaluate whether MACE was reduced by statin treatment. MACE was defined by occurrence of any of the following for the purpose of this study: cardiovascular death, all-cause death, new myocardial infarction, recurrence of unstable angina, and new cerebrovascular events. For CMVD, we restricted our analysis to patients who had CMVD without obstructive coronary artery disease (N=925).

### 2.1. Study Population

**Figure 1:**
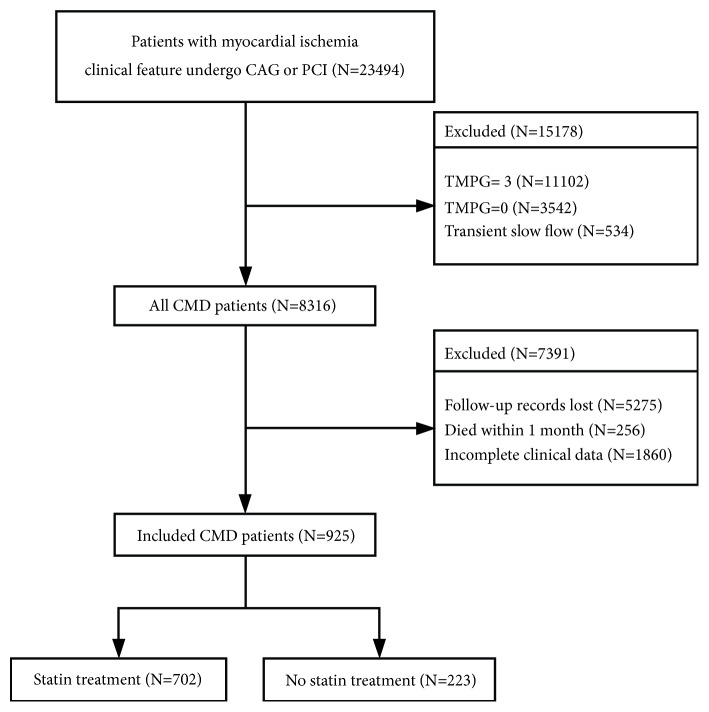
The criteria of inclusion and exclusion.

Of the 23,494 patients who met the inclusion criteria, 22,568 patients were excluded for the following reasons: 11,102 patients had an estimated TMPG of 3, 3,542 patients had completely blocked coronary arteries, 534 had transient slow blood flow during PCI, 5,275 patients lost their followup records, 256 patients died within 1 month of CAG or PCI, and 1,860 patients had missing followup clinical data (Figure 1). A total of 925 subjects were included.

### 2.2. Participant and Public Involvement

Before launching this study, we held a forum to explore patient priorities regarding CMVD prevention and experiences with medications, which helped inform the study design. This study will be used to educate and inform CMVD patients of progress of the study. Participants will also receive an annual update on the progress of the study via a followup.

### 2.3. Subject Characteristics

Demographic data and clinical variables related to age, sex, smoking history, age, body mass index (BMI), hypertension, diabetes mellitus, chronic kidney disease, hyperlipidemia, New York Heart Association classification (NYHA) III/IV, atrial fibrillation/atrial flutter, PCI history, total cholesterol (TC), triglyceride (TG), high-density lipoprotein (HDL-C), low-density lipoprotein (LDL-C), glucose (Glu), blood urea nitrogen (BUN), creatinine, alanine aminotransferase (ALT), aspartate aminotransferase (AST), CK-MB, cTnT, NT-proBNP, white blood cell count, hemoglobin, platelet count, uric acid, left ventricular ejection fraction (LVEF) percentage, blood systolic pressure, blood diastolic pressure, heart rate, renin-angiotensin-aldosterone system (RAAS), calcium channel blockers (CCB), *β*-blockers, antisterones, diuretics, aspirin, clopidogrel/ticagrelor, and statins were collected retrospectively from the electronic clinical research database at the Heart Center, ZhuJiang hospital, China. TMPG was used to assess coronary microvascular function. The results presented here were part of a larger study. Epidemiology and cognitive, physical, and psychological status for this sample were described elsewhere (Table 1).

### 2.4. Statistical Analysis

Distributions and categories were examined, and categories with small sample sizes and skewed distributions were noted. Continuous variables were expressed as mean ± standard deviation (SD) and compared using Student's t-test or Mann-Whitney U test, as appropriate. Categorical variables were compared between the groups using chi-squared or Fisher's exact test. Cox and Kaplan-Meier survival analysis were used to examine the association between statin treatment and MACE in patients with CMVD. Furthermore, we used subgroups analysis to compare the hazard ratio (HR) between statin or no statin treatment in patients with CMVD with various cointervention factors such as smoking history, diabetes, hypertension, chronic kidney disease, dyslipidemia, PCI history, heart failure, etc. IBM SPSS version 22.0 was used to analyze the data. Categories were meaningfully combined when indicated. All statistical tests were two-sided, and significance was set at* P*<0.05.

## 3. Results

### 3.1. Study Cohort Characteristics

This study consisted of 925 patients who underwent an elective coronary angiography in our center (mean age = 61.71 ± 11.31 years). Baseline characteristics of the study cohort are summarized in Table 1. There were fewer males (n=364; 39.4%) than females (n=561; 60.6%) in the study. Moreover, there were more smokers or ex-smokers in the nonstatin group (n=419; 45.3%) compared to the statin group (n=62; 6.7%, P<0.001). Furthermore, more patients had hypertension and/or diabetes mellitus in the nonstatin group compared to the statins group. There were more patients with PCI history in the nonstatin group (n=436; 47.1%) compared to the statin group (n=87; 9.4%, P<0.001).

### 3.2. Primary Outcomes

925 CMVD cases were chosen from 23,494 patients in this study. Using univariate analysis, being female (HR, 0.71; 95% CI, 0.58-0.87;* P=*0.001), smoking history (HR, 0.53; 95% CI, 0.42-0.65;* P<*0.001), hypertension (HR, 0.57; 95% CI, 0.46-0.70;* P<*0.001), chronic kidney disease (HR, 0.47; 95% CI, 0.35-0.64;* P<*0.001), TG (HR, 1.10; 95% CI, 1.02-1.19;* P=*0.011), creatinine (HR, 1.00; 95% CI, 0.99-1.02;* P=0.034*), cTnT (HR, 0.98; 95% CI, 0.97-0.99;* P<*0.001), platelet count (HR, 0.99; 95% CI, 0.99-0.99;* P=*0.009), heart rate (HR, 0.99; 95% CI, 0.98-1.00;* P=*0.005), RAAS (HR, 1.24; 95% CI, 1.02-1.52;* P=*0.031), CCB (HR, 1.32; 95% CI, 1.01-1.74;* P=*0.044), *β*-blockers (HR, 1.69; 95% CI, 1.38-2.07;* P<*0.001), aspirin (HR, 1.50; 95% CI, 1.18-1.91;* P=*0.001), clopidogrel/ticagrelor (HR, 1.99; 95% CI, 1.45-2.72;* P<*0.001), and statins (HR, 0.42; 95% CI, 0.32-0.56;* P<*0.001) were significantly associated with overall survival in patients with CMVD. Using multivariate analysis, NYHA III/IV (HR, 1.44; 95% CI, 1.03-2.01;* P=*0.031), PCI history (HR, 3.69; 95% CI, 2.57-5.31;* P<0.001)*, TG (HR, 1.15; 95% CI, 1.06-1.26;* P=*0.001), creatinine (HR, 1.00; 95% CI, 1.00-1.01;* P<*0.001), cTnT (HR, 0.98; 95% CI, 0.96-0.99;* P*<0.001), heart rate (HR, 0.98; 95% CI, 0.97-0.99;* P=*0.001), *β*-blockers (HR, 0.68; 95% CI, 0.51-0.91;* P=*0.008, aspirin) (HR, 0.38; 95% CI, 0.24-0.61;* P<0.001)*, and statins (HR, 0.33; 95% CI, 0.19-0.60;* P<0.001)* were significantly associated with overall survival in patients with CMVD (Table 2).

For all analyses, patients were divided into two groups: statin (N=702, 75.9%) or nonstatin (N=223, 24.1%). All basic information pertaining to the two groups is summarized in Table 1. Rates of MACE were categorized according to cause of death, such as death from all-causes, cardiac-related death, recurrent stroke, recurrent myocardial infarction, and unstable angina.

**Figure 2:**
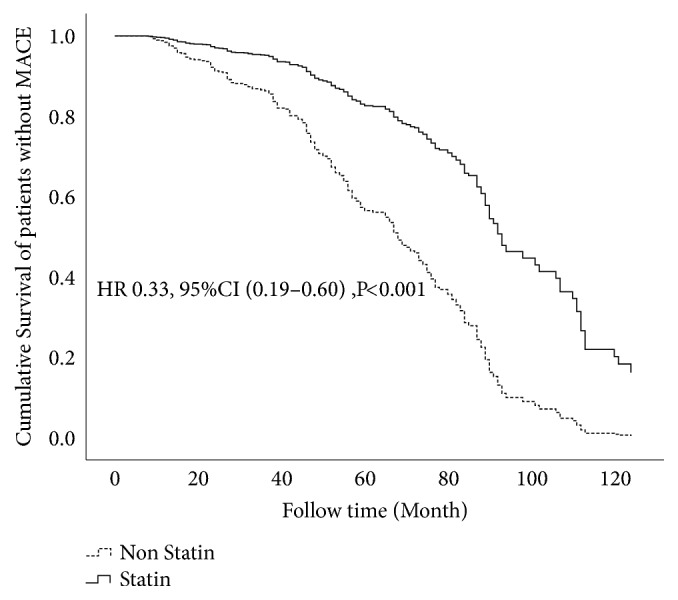
Cox multivariate analysis of cumulative survival of patients with statin treatment and nonstatin treatment.

Univariate analysis and multivariate analysis results showed that statin treatment was associated with better outcomes in patients with CMVD. We examined differences between the statin and nonstatin groups. Adjusted for NYHA III/IV, PCI history, *β*-blockers, aspirin, statins, creatinine, cTnT, and heart rate, Cox multivariate analysis showed that statins reduced MACE events in CMVD patients (HR 0.33, 95%CI 0.19-0.60,* P*<0.001). Cox survival analysis curves are shown in Figure 2.

### 3.3. Subgroup Analysis Outcome

**Figure 3:**
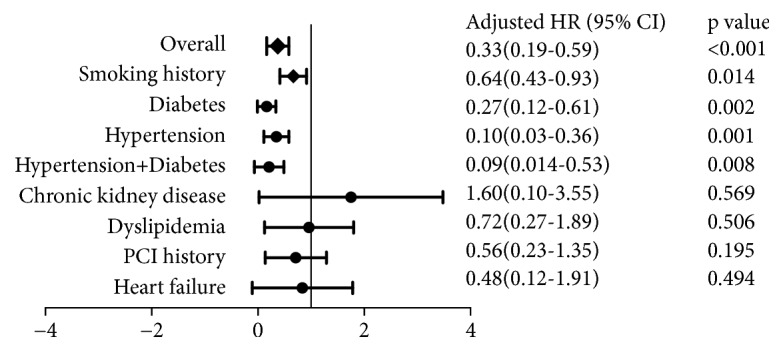
Subgroup analysis to evaluate benefits of statin treatment in patients with CMVD with various co-intervention factors.

Subgroup analysis was used to evaluate benefits of statin treatment in patients with CMVD with various cointervention factors such as hypertension, DM, etc. In subgroup analysis, statins were associated with decreased MACE in patients overall (HR, 0.33; 95% confidence interval, 0.19-0.59;* P*<0.001) and in patients with smoking history (HR, 0.64; 95% CI, 0.43-0.93;* P=*0.014), diabetes (HR, 0.27; 95% CI, 0.12-0.61;* P=*0.002), hypertension (HR, 0.10; 95% CI, 0.03-0.36;* P=*0.001), and hypertension and diabetes (HR, 0.09; 95% CI, 0.014-0.53;* P=*0.008) (Figure 3).

## 4. Discussion

In this study, we found that statin treatment on admission was associated with positive outcomes in patients with coronary microvascular dysfunction. Furthermore, using univariate analysis and multivariate analysis adjusted for various factors of disease HR, statins were independently associated with decreased long-term morbidity and mortality in hospitalized coronary microvascular dysfunction patients. In the present study, we showed that statin treatment is associated with reduced all-cause mortality for a long-term followup period of greater than 10 years. Furthermore, statins were independently related to HR in the CMVD cohort.

CMVD without epicardial coronary stenosis or occlusion and cardiomyopathy were mostly caused by risk factors of coronary heart disease which was partially reversible. Therefore, providing guidance to patients to eliminate these risk factors at early stages of CMVD may prove beneficial. Slow flow phenomenon is a characteristic of CMVD which refers to patients with recurring chest pain with normal or less than 50% narrowed subpericardial vessels as determined by coronary angiography [12].

The mechanisms of CMVD pathology are unknown, but many studies have shown that multiple factors play significant roles in development of CMVD, including microvascular disease, vascular endothelial dysfunction, inflammatory reaction, and abnormal blood rheology [13].

TMPG was predictive of CMVD. Elevated evidence showed that patients with low TMPG are at high risk for MACE and subsequent increased mortality risk. TMPG was a useful tool for measurement of coronary blood flow [14–16].

Recent studies have found that anatomical characteristics of coronary arteries and thickness of subpericardial fat correlated with occurrence of CMVD [17, 18]. Multiple clinical studies have shown that most risk factors for coronary atherosclerosis were also risk factors for CMVD, such as smoking, being male, hyperhomocysteinemia, abnormal glucose tolerance, diabetes, hypersensitive CRP, hyperlipidemia, and other independent predictors of CMVD [19].

The mechanisms by which statins could decrease MACE in patients with CMVD are unclear. We hypothesized that statins play a significant role in decreasing MACE by anti-inflammatory activity, improvement of endothelial cell function, and reduced oxidative stress. Statins can inhibit expression of NF-*κ*B and macrophage tissue factor, increase nitric oxide in endothelial cells, and inhibit chemotaxis and platelet aggregation of inflammatory cells into plaques, thus improving vascular endothelial function [20]. Moreover, statins can reduce activity of C-reactive protein-mediated mononuclear inflammatory response, regulate signaling pathways such as NF-*κ*B and p38 mitogen activated protein kinase, reduce expression of chemokines and adhesion molecules, and inhibit formation of proinflammatory cytokines such as interleukin-1, interleukin-6, and TNF-*α*. As a result, statins reduce the inflammatory response, inhibit plaque formation, stabilize plaques, and reduce plaque rupture [21, 22].

Mechanisms contributing to the association of statin treatment and reduced all-cause mortality in patients with CMVD may include a combination of oxidative stress reduction and inflammatory response, in addition to decreased LDL cholesterol. Increased plasma LDL concentration results in retention in the endothelial layer of blood vessels, leading to oxidative modification to form lipid peroxides, phospholipid compounds, and carbonyl groups. These lipid molecules could induce macrophages and blood vessel wall cells to produce cell adhesion molecules, chemical factors, and inflammatory mediators and activate the injury-response process for damaged vascular endothelium [23, 24]. In addition, apolipoproteins can also be modified, resulting in autoantigenicity, activation of T cells, antigen-specific immune responses to promote inflammatory cell aggregation in atheromatous plaques, exacerbation of lipid accumulation, worsened endothelial dysfunction, and smooth muscle proliferation. Consequently, antioxidant and anti-inflammatory effects may explain why all-cause mortality was reduced in the statin group.

### 4.1. Strengths and Limitations

Strengths of this study include engagement of a large number of patients from a large restrospective cohort study from an electronic database. In addition, the effects of statin treatment on MACE in patients with CMVD had not been previously evaluated.

Our analysis had several limitations. First, lack of a clear diagnostic standard for coronary microvascular dysfunction may have reduced the accuracy of the inclusion and exclusion standards. Second, medical treatment of coronary microvascular dysfunction can help accelerate recovery of blood flow reserve, but the effect on prognosis remains uncertain. Third, baseline characteristics between the two groups could not be completely compared. Fourth, there was no randomized controlled trial available for subgroup analysis. Because of unavailability of combined MACE outcomes data in the original studies, we were unable to include these in our analysis. Finally, the cross-sectional nature of this study precludes any inference about cause and effect relationship. Longitudinal studies are needed to address causality.

## 5. Conclusion

In this study, we found that statin treatment was associated with reduced MACE in CMVD patients over a long-term period (more than 10 years). Further prospective studies are needed to demonstrate the benefits of long-term statin treatment.

## Figures and Tables

**Table 1:** Baseline characteristics of the study population and comparison between statin treatment and non-statin treatment.

	ALL	Statin	Non-statin	P value
Age	61.71 ± 11.31	63.12 ± 12.71	61.49 ± 11.08	0.296
Female sex n (%)	561 (60.6)	178 (12.4)	383 (41.4)	<0.001
Smoking history n (%)	481 (52.0)	62 (6.7)	419 (45.3)	<0.001
BMI	24.61 ± 3.76	24.19 ± 4.11	24.69 ± 3.69	0.373
Hypertension n (%)	266 (28.8)	34 (3.7)	232 (25.1)	<0.001
Diabetes mellitus n (%)	138 (14.9)	16 (1.7)	122 (13.2)	<0.001
CKD n (%)	99 (10.7)	17 (1.8)	82 (8.9)	0.088
Hyperlipemia n (%)	166 (17.9)	23 (2.5)	143 (15.5)	0.001
NYHA III/IV n (%)	118 (12.8)	15 (1.6)	103 (11.1)	0.002
AF/ AFL n (%)	19 (2.1)	2 (0.2)	17 (1.8)	0.162
PCI history n (%)	523 (56.5)	436 (47.1)	87 (9.4)	<0.001
CABG N (%)	1 (0.1)	1 (0.1)	0 (0)	0.076
Lipid profile				
TC	4.73 ± 1.30	4.42 ± 1.24	4.79 ± 1.30	0.013
TG	1.74 ± 1.41	1.55 ± 1.03	1.77 ± 1.47	0.090
HDL-C	1.17 ± 0.32	1.16 ± 0.33	1.17 ± 0.32	0.740
LDL-C	2.95 ± 1.03	2.79 ± 0.99	2.97 ± 1.04	0.136
Glucose	6.30 ± 2.72	6.51 ± 2.83	6.25 ± 2.70	0.429
BUN	6.75 ± 3.77	8.30 ± 5.63	6.44 ± 3.19	0.001
Creatinine	121.72 ± 86.25	140.31 ± 104.53	118.00 ± 81.71	0.030
ALT	53.00 ± 107.31	87.31 ± 205.74	46.26 ± 72.44	0.046
AST	86.98 ± 175.80	87.41 ± 190.23	86.9 ± 173.02	0.989
CK-MB	55.20 ± 98.55	48.14 ± 106.52	56.47 ± 97.12	0.495
cTnT	6.97 ± 14.15	7.59 ± 15.22	6.87 ± 13.98	0.701
NT-proBNP	877.80 ± 1135.45	965.80 ± 1187.42	863.00 ± 1128.68	0.631
WBC	9.70 ± 3.44	9.91 ± 3.91	9.66 ± 3.34	0.514
Hemoglobin	128.89 ± 19.49	123.17 ± 22.41	130.06 ± 18.64	0.002
Platelet count	230.55 ± 70.56	230.80 ± 85.19	230.50 ± 67.26	0.972
Uric acid	384.77 ± 124.04	388.57 ± 123.12	384.05 ± 124.35	0.767
LVEF%	46.87 ± 10.60	45.38 ± 12.24	47.10 ± 10.33	0.297
Blood pressure				
Diastolic pressure	78.18 ± 14.75	76.72 ± 14.84	78.41 ± 14.74	0.359
Systolic pressure	129.25 ± 25.24	125.24 ± 19.65	129.87 ± 25.95	0.056
Heart rate	74.30 ± 16.89	77.16 ± 19.49	73.73 ± 16.28	0.073
Medication				
RAAS n (%)	482 (52.1)	166 (17.9)	316 (34.2)	<0.001
CCB n (%)	802 (86.7)	205 (22.2)	597 (64.5)	0.009
*β*-blocker n (%)	422 (45.6)	156 (16.9)	266 (28.8)	<0.001
Antisterone n (%)	742 (80.2)	195 (21.1)	547 (59.1)	0.002
Diuretic n (%)	678 (73.3)	174 (18.8)	504 (54.5)	0.067
Aspirin n (%)	273 (29.5)	182 (19.7)	91 (9.8)	<0.001
Clopidogrel/ticagrelor	192 (20.8)	156 (16.9)	36 (3.9)	<0.001
Follow time (Month)	63.08 ± 35.48	69.43 ± 37.24	61.07 ± 34.68	0.003
MACE n (%)	396 (42.8)	56 (6.1)	340 (36.8)	<0.001

BMI, Body Mass Index; CKD, chronic kidney disease; AF, atrial fibrillation; AFL, atrial flutter; PCI, percutaneous coronary intervention; CABG, coronary artery bypass grafting; TC, total cholesterol; TG, triglyceride; HDL-C, high-density lipoprotein cholesterol; LDL-C, low-density lipoprotein cholesterol; RAAS, renin-angiotensin-aldosterone system; CCB, calcium channel blockers; BUN, urea nitrogen; WBC, white blood cell count; NYHA, New York Heart Association classification; Glu, glucose; ALT, alanine aminotransferase; AST, aspartate aminotransferase; LVEF, left ventricular ejective fraction; RAAS, renin-angiotensin-aldosterone system (RAAS); MACE, Major Adverse Cardiovascular Events

**Table 2:** Results of univariate and multivariate Cox proportional hazards model regression analysis of MACE.

Variables	Univariate analysis		Multivariate anaysis	
	HR (95% CI)	P value	HR (95% CI)	P value
Age	1.01 (0.99-1.02)	0.225		
Femal sex n (%)	0.71 (0.58-0.87)	0.001		
Smoking history n (%)	0.53 (0.42-0.65)	<0.001		
BMI	1.02 (0.97-1.06)	0.463		
Hypertension n (%)	0.57 (0.46-0.70)	<0.001		
Diabetes mellitus n (%)	0.71 (0.55-0.92)	0.010		
Chronic kidney disease n (%)	0.47 (0.35-0.64)	<0.001		
Hyperlipemia n (%)	0.56 (0.45-0.71)	<0.001		
NYHA III/IV n (%)	0.54 (0.40-0.72)	<0.001	1.44 (1.03-2.01)	0.031
AF /AFL n (%)	0.19 (0.09-0.39)	<0.001		
PCI history n (%)	0.31 (0.24-0.40)	0.310	3.69 (2.57-5.31)	<0.001
Lipid profile				
TC	1.00 (0.92-1.09)	0.973		
TG	1.10 (1.02-1.19)	0.011	1.15 (1.06-1.26)	0.001
HDL-C	0.93 (0.67-1.31)	0.698		
LDL-C	0.99 (0.89-1.10)	0.805		
Glucose	0.95 (0.91-1.00)	0.053		
BUN	0.97 (0.94-1.01)	0.160		
Creatinine	1.00 (0.99-1.02)	0.034	1.00 (1.00-1.01)	<0.001
ALT	0.99 (0.99-1.00)	0.213		
AST	0.99 (0.99-1.00)	0.488		
CK-MB	0.99 (0.98-1.00)	0.235		
cTnT	0.98 (0.97-0.99)	<0.001	0.98 (0.96-0.99)	<0.001
NT-proBNP	1.00 (1.00-1.00)	0.015		
WBC	0.98 (0.95-1.01)	0.132		
Hemoglobin	1.01 (1.00-1.01)	0.072		
Platelet count	0.99 (0.99-0.99)	0.009		
Uric acid	1.00 (0.99-1.00)	0.700		
LVEF%	1.00 (0.99-1.01)	0.949		
Systolic pressure	1.01 (0.99-1.01)	0.095		
Diastolic pressure	0.99 (0.08-1.00)	0.123		
Heart rate	0.99 (0.98-1.00)	0.005	0.98 (0.97-0.99)	0.001
RAAS n (%)	0.81 (0.66-0.98)	0.031		
CCB n (%)	0.76 (0.58-0.99)	0.044		
*β*-blocker n (%)	0.59 (0.48-0.72)	<0.001	0.68 (0.51-0.91)	0.008
Antisterone n (%)	0.89 (0.64-1.23)	0.471		
Diuretic n (%)	1.01 (0.75-1.37)	0.931		
Aspirin n (%)	0.67 (0.52-0.85)	0.001	0.38 (0.24-0.61)	<0.001
Clopidogrel/ticagrelor	0.50 (0.37-0.69)	<0.001		
Statin	0.42 (0.32-0.56)	<0.001	0.33 (0.19-0.60)	<0.001

BMI, Body Mass Index; CKD, chronic kidney disease; AF, atrial fibrillation; AFL, atrial flutter; PCI, percutaneous coronary intervention; CABG, coronary artery bypass grafting; TC, total cholesterol; TG, triglyceride; HDL-C, high-density lipoprotein cholesterol; LDL-C, low-density lipoprotein cholesterol; RAAS, renin-angiotensin-aldosterone system; CCB, calcium channel blockers; BUN, urea nitrogen; WBC, white blood cell count; NYHA, New York Heart Association classification; ALT, alanine aminotransferase; AST, aspartate aminotransferase; LVEF, left ventricular ejective fraction; RAAS, renin-angiotensin-aldosterone system (RAAS); MACE, Major Adverse Cardio.

## Data Availability

The electronic spreadsheet data used to support the findings of this study are available from the corresponding author upon request. Additionally, data used to support the findings of this study are also included within the article.
